# Association between Different Types of Exercise and Intake of Nutrients including Carbohydrate, Fat, Protein, and B Vitamins in Young Adults

**DOI:** 10.3390/nu15040806

**Published:** 2023-02-04

**Authors:** Jing Zhang, Jiangang Chen, Xuemei Sui, Clemens Drenowatz, Qirong Wang

**Affiliations:** 1 School of Physical Education, Shaanxi Normal University, Xi’an 710119, China; 2 School of Physical Education and Sports, Beijing Normal University, Beijing 100875, China; 3 Department of Exercise Science, Arnold School of Public Health, University of South Carolina, Columbia, SC 29202, USA; 4 Division of Sport, Physical Activity and Health University of Education Upper Austria, Kaplanhofstraße 40, 4020 Linz, Austria; 5 Sports Nutrition Center, National Institute of Sports Medicine, Beijing 100029, China

**Keywords:** exercise types, nutrient intakes, B vitamins, young adults

## Abstract

Purpose: This cross-sectional study aimed to investigate the association between different types of exercise and nutrient intake to provide a basis for promoting the overall health of young adults. Methods: A total of 427 young adults (217 women) aged 21 to 35 were recruited. Participants self-reported time spent (min/week) in endurance exercise, resistance exercise, sports, walking, and other structured physical activity (PA). Nutrient intake was determined via telephone-administered 24 h recalls. Results: Resistance exercise was positively associated with intake of protein, vitamins B2, B3, B5, B6, and B12 and the percentage of total calories from protein (PCT-PRO), and negatively associated with the percentage of total calories from carbohydrate (PCT-CHO) (*p* < 0.05). Time spent in aerobic exercise was positively associated with fiber, pectin, and vitamin B6 intake, and negatively associated with PCT-PRO (*p* < 0.05). Time spent exercising was negatively associated with fiber and pectin intake (*p* < 0.05). Time spent performing other structured PA was positively associated with pectin intake (*p* < 0.05). Participants’ total exercise time was positively associated with intake of vitamins B2, B5, B12, and PCT-Fat, PCT-PRO, and negatively associated with PCT-CHO (*p* < 0.05). Conclusion: The results showed an association between various exercise types and specific nutrients. It may be worthwhile to point out the negative association of exercise with CHO intake, which may need to be examined more closely in active young adults. In addition, the supplementation of B vitamins and pectin may be beneficial for their exercise performance and post-exercise recovery.

## 1. Introduction

A healthy lifestyle can provide many benefits; for example, reducing the risk of chronic disease [1], promoting mental health [2], improving the quality of life for people with cancer [3], and reducing the incidence of age-related diseases [4,5]. As the two pillars on which a healthy lifestyle is based, physical activity and dietary patterns are among the hot topics of research that have long received widespread attention [6]. Several systematic meta-analyses have shown the benefits of combined exercise and diet interventions for weight control, muscle mass and strength, and bone health [7,8,9]. The role of exercise combined with diet in improving or maintaining cognitive function in older adults has also been demonstrated in several large randomized controlled trials [10,11]. Some research suggested that physical activity may share a common neurocognitive platform with eating behaviors [12,13]. However, exercise and diet may not work independently, but are rather associated with each other. Therefore, there is a necessity for the more extensive research on the relationship between exercise and dietary nutrition.

Previous studies have shown that the eating habits and nutritional choices of athletes engaged in different sports may vary. Spendlove et al. [14] reported that athletes who engage in long-term bodybuilding training had daily carbohydrate intakes below the recommended standard, protein intakes above the standard, and intakes of vitamins B1, B2, B3, and B12 even above the upper tolerable limit. A study of amateur marathoners indicated that athletes’ intakes of fiber, vitamins D and E were below the recommended levels, while vitamins A and C were above the daily reference values [15]. Knez et al. also noted in their study that over 60% of ultra-endurance triathletes use vitamin supplements, with vitamins C and E being the most prevalent [16]. In addition to the athlete population, studies on the general population have had similar findings [17,18]. After 9 weeks of high-intensity interval training, young adults aged 21–24 years were determined to autonomously regulate their dietary choices and intake, resulting in a healthier diet [19]. Several previous prospective studies have shown that exercise appeared to change the dietary preferences and total intake of the average young person, and could lead to healthier nutritional choices, such as reduced snacking and increased intake of vegetables and fruits [20,21]. Guezennec et al. [22] investigated 10,373 young people and determined that physical activity levels were not related to calcium intake levels.

To date, several studies on athletes have reported the association between different sports and macronutrients and micronutrients [14,23]. However, most studies involving the general population have only explored the association of physical activity with eating behavior in a broad sense, rather than examining the association between specific types of exercise and nutrients [21]. More importantly, to our knowledge, there are even fewer studies involving micronutrient intake, such as B vitamins, fiber, and pectin. Micronutrients such as B vitamins, fiber, and pectin were not only determined to play an important role in improving performance, preventing injury, and promoting recovery from exercise fatigue [24,25], but were also essential in establishing proper dietary patterns and promoting overall health [26,27]. However, for the general population, whether specific types of exercise such as aerobic and resistance exercise also influence eating behavior and nutritional intake is unknown. Therefore, this study aimed to investigate the association between different types of self-selected exercise and nutrient intakes including carbohydrates, fats, proteins, and B vitamins with the ultimate goal of characterizing the macro- and micro-nutrient intakes of young adults participating in different types of exercises.

## 2. Materials and Methods

### 2.1. Participants

The data analyzed in the present study were derived from baseline data of a large longitudinal observational study, which was designed to investigate the energy balance of young adults aged over 12 months. The details of the energy balance study have been published and described in full previously [28]. Briefly, 430 young adults (218 women and 212 men) aged 21 to 35 with a body mass index between 20 and 35 kg/m^2^ were recruited. Participants in this age group were selected for the energy balance study due to the fact that they were at risk for reduced metabolic rate and increased body fat percentage [29]. Participants were asked to use the telephone for the diet recall interview. Individuals who had major acute and chronic medical conditions, used diet pills or had a history of depression, panic or anxiety were excluded from this study. All participants provided written informed consent at the time of baseline data collection. The protocol for the energy balance study was approved by the University of South Carolina Institutional Review Board and was conducted in accordance with the Declaration of Helsinki [30].

### 2.2. Anthropometric Measurements

Measurements of height, weight, and body composition were obtained during the second phase of the baseline test. Body height and weight were measured with a stadiometer (Model S100, Ayrton Corp., Prior Lake, MN, USA) and an electronic scale (Model 500 KL, McCook, IL, USA), and the measurements were recorded to the nearest 0.1 cm and 0.1 kg, respectively. Height and weight were measured three times, and the average was calculated and recorded. Body mass index (BMI, kg/m^2^) was calculated using the average height and weight. Total fat and total lean mass were measured with a dual X-ray absorptiometry (DXA) scanner (GE Healthcare model 8743, Waukesha, WI, USA). Body fat percentage (%) was determined using total fat mass divided by body weight. All anthropomorphic measurements were conducted while participants were wearing surgical scrubs and barefoot.

### 2.3. Physical Activity and Exercise Participation

Each participant was provided with a tri-axial accelerometer (SenseWear^®^ Mini Armband, BodyMedia Inc., Pittsburgh, PA, USA) and was asked to wear it for 10 days to monitor total daily energy expenditure. The level of physical activity was determined according to SenseWear’s professional algorithm (version 7.0 BodyMedia Inc., Pittsburgh, PA). The SenseWear^®^ Mini Armband has been shown to provide valid estimates of energy expenditure and physical activity levels in adults [31].

The different types of exercise were self-reported by participants. The specific types of exercise included upper-body resistance exercise, lower-body resistance exercise, sports, cycling, running, swimming, aerobic/group exercises, walking, and other structured physical activities. The frequency of these specific exercises was reported in days per week and the duration of the exercise was reported in the form of minutes per day. Subsequently, similar types of exercises were grouped, specifically running, cycling, and swimming were categorized as endurance exercises, and upper-body resistance training and lower-body resistance training were categorized as resistance training. Finally, the five main types of exercise—endurance exercise, resistance exercise, sports, walking, and other structured PA—were determined in the form of minutes performed per week (min/week). Considering that the median weekly exercise time for most exercise types was 40 min and the median total weekly exercise time was 240 min, single types of exercise time were categorized as < or >40 min and total exercise time was categorized as < or >240 min. The classification criteria for total exercise time were higher than the PA recommendation of 150 min/week of MVPA + resistance exercise in order to account for the overall high level of exercise time commitment of participants in the study and the fact that too low of a cut point may obscure possible associations. 

### 2.4. Measurement of Nutrient Intakes

Nutrient intake was assessed by calculating participants’ self-reported food consumption. Participants underwent a 10- to 15-min training session during the baseline testing to learn the proper use of food portion visualization (FPV) to assess the portion size of common foods. This training featured real-life plates, glasses, and food models combined with hands-on experiential communication. Following the training, data on food consumption were collected via multiple 24 h dietary recall interviews that were administered on non-consecutive days via telephone during the time the participants were wearing the SenseWear^®^ Mini. The validity of this method has been examined in previous studies [32]. To reduce the potential for bias, the time of the telephone interviews was chosen randomly and the telephone numbers were unfamiliar to the interviewers. The 24 h dietary recall interview was completed by a registered dietitian with specialized training and more than 6 years of experience. Participants needed to have data from at least two 24 h dietary recall interviews in order for these data to be included in the analysis. The Nutrient Data System for Research software (NDSR Version 2012), licensed from the Nutrition Coordinating Center (NCC) at the University of Minnesota, was used to calculate macro- and micro-nutrient intakes. With a database of over 19,000 foods and access to information from over 120 nutrients, the NDSR is one of the most advanced research software that allows for 24 h recall interviews. After obtaining the intake of fat, carbohydrates, and protein, the calories provided by the three macro-nutrients were calculated and then divided by the total calorie intake, respectively, to obtain the percentage of total calories from fat (PCT-Fat), percentage of total calories from carbohydrates (PCT-CHO) and percentage of total calories from protein (PCT-PRO). The dietary reference intakes for B vitamins [33] and macro-nutrients [34] in this study were obtained from the guidelines published by the Food and Nutrition Board, Institute of Medicine in 1998 and 2002, respectively.

### 2.5. Statistical Analysis

The normality of the data was tested using the Kolmogorov–Smirnov test. Basic descriptive statistics are presented in the form of mean ± standard deviation for normally distributed data, median and interquartile range for non-normally distributed data, and frequency and percentage for categorical data. Multiple linear regression was used to determine the associations between different types of self-selected exercise and nutrient intake. Linear regression models controlled for sex, age, ethnicity, employment status, and marital status. All variables were standardized for comparison at the same scale before linear regression analysis was performed. Binary logistic regression was used to explore the associations between exercise time and whether nutrient intake met DRI criteria. Logistic regression models controlled for sex, age, and ethnicity. According to the calculation of PASS11.0 (NCSS, LLC., Kaysville, UT, USA), the statistical power of both linear and logistic regression models reached 85%. A *p*-value < 0.05 was considered statistically significant. SPSS 23.0 software (IBM Corp., Armonk, NY, USA) was used for statistical analysis.

## 3. Results

From the 430 participants recruited, a total of 427 participants (50.8% women) provided valid information on exercise time and dietary nutrient intake. Three participants did not provide information on their dietary intake and were excluded from this study. Table 1 shows the baseline characteristics of the participants. The mean age of participants was 27.65 ± 3.78 years and 66.5% of participants were European American, 52.5% were employed for wages, and 32.6% were married. The mean height of men and women was 177.84 ± 6.98 and 164.57 ± 6.49 cm, respectively. Their BMI was above the threshold of 25. Both fat mass and body fat percentage were higher in women than in men, and lean mass was higher in men than in women. Women spent more time performing light PA, while time spent performing MVPA was higher in men.

Table 2 shows that the participants of different EX type spent time in various ways. The exercise time that most of the people spent in sports and other EX was 0 min. Exercise time of 40 min per week was considered the cutpoint. Most people spent less than 40 min on exercises of resistance, sports, walking and other EX in addition to aerobic EX. 

The total EX median time of 240 min per week was considered the cut point. Table 3 shows that intake of carbohydrates, protein, vitamin B2, and vitamin B3/niacin met the DRI criteria for more than 90% of the participants whose EX time was more than 240 min. Slightly fewer participants met the DRI criteria for vitamin B1, B6, and B12 intake at just over 80% while only approximately half of the participants’ vitamin B5/pantothenic acid and vitamin B9/folate intakes met the DRI criteria. Fiber was the nutrient with the lowest percentage (9.1%) of participants’ intake meeting DRI standards of all nutrients.

Table 4 shows that participants’ time spent in resistance exercise was positively associated with intake of protein, vitamins B2, B3, B5, B6, and B12 and PCT-PRO (*β* = 0.157, *β* = 0.138, *β* = 0.141, *β* = 0.161, *β* = 0.143, *β* = 0.149, *β* = 0.191, *p* < 0.05), and negatively associated with PCTCHO (*β* = −0.166, *p* < 0.05). Time spent in aerobic exercise was positively associated with fiber, pectin, and vitamin B6 intake (*β* = 0.129, *β* = 0.115, *β* = 0.101, *p* < 0.05), and negatively associated with PCT-PRO (*β* = 0.130, *p* < 0.05). Time spent partaking in sports activities was negatively associated with fiber and pectin intake (*β* = −0.140, *β* = −0.194, *p* < 0.05). Time spent performing other structured PA was positively associated with pectin intake (*β* = 0.131, *p* < 0.05). Participants’ total exercise time was positively associated with intake of vitamins B2, B5, B12 and PCT-Fat, PCT-PRO (*β* = 0.138, *β* = 0.155, *β* = 0.130, *β* = 0.116, *β* = 0.126, *p* < 0.05), and negatively associated with PCT-CHO (*β* = −0.142, *p* < 0.05).

Table 5 shows that 49.4% of participants with more than 40 min of weekly aerobic exercise had 49.4% (OR = 0.506; 95%CI 0.285 to 0.899) fewer odds of meeting vitamin B12 DRI criteria compared to those with less than 40 min of weekly aerobic exercise. Participants with more than 40 min of walking time had 53.9% (OR = 0.461; 95%CI 0.248 to 0.856) fewer odds of meeting vitamin B6 DRI criteria compared to those with less than 40 min of walking time. Binary logistic regression also showed that participants with more than 240 min of weekly total exercise time had a 2.023-fold (OR = 3.023; 95%CI 1.403 to 6.514) and 0.616-fold (OR = 1.616; 95%CI 1.061 to 2.462) increase in the odds of consuming fiber and vitamin B5 that met DRI standards, respectively, compared to participants with less than 240 min of total exercise time.

## 4. Discussion

This study explored the association between different self-selected exercise types and intake of various macro- and micro-nutrients in young adults. The main finding of this study was that the time spent in resistance exercise and total exercise time were positively associated with most B-vitamin intake and the percentage of total calories from protein (PCT-PRO) and negatively associated with the percentage of total calories from carbohydrates (PCT-CHO). We also observed that the time spent in aerobic exercise was positively associated with fiber and pectin intake and negatively associated with PCT-PRO. Furthermore, after dividing the time spent on exercise according to the median, we determined that aerobic exercise and walking for periods greater than 40 min were associated with a lower likelihood of meeting vitamin B12 and B6 Reference Intake (DRI) requirements. On the other hand, we determined that participants with more than 240 min of weekly total exercise time had higher odds of meeting DRI criteria for fiber and vitamin B5 intake. These results suggest that the type of exercise engaged in is strongly associated with the intake of macro-nutrients and B vitamins. 

In this study, time spent in resistance exercise was most closely associated with the intake of two major macronutrients: carbohydrate and protein. We observed a positive association between the time spent in resistance exercise and protein intake as well as the percentage of total calories from protein. This is consistent with previous findings in bodybuilders [35]. Bodybuilders tend to consume amounts of protein that are higher than recommended in order to meet the needs of muscle development [36]. For the general population, there is also growing evidence that a high-protein diet is beneficial for increasing lean body mass and reducing fat [37]. Because of the many possible benefits of consuming high biological value protein before and after resistance exercise [38], people who regularly participate in resistance training are likely to consume more protein to achieve better workout results. In addition, we observed that the time spent in resistance exercise was negatively associated with the percentage of total calories obtained from carbohydrates. However, this association is not reflected in the absolute intake of carbohydrates. This can, therefore, only suggest that as protein intake increases, carbohydrate intake decreases in relative terms, thus showing an increase in the percentage of total calories from protein and a decrease in the percentage of total calories from carbohydrates, which may be related with the relative stability of total energy intake for a person. However, even though this high-protein, low-carbohydrate diet may have many potential benefits for weight loss and body shaping [39], it should not be overlooked that muscle glycogen is very important for resistance exercise [40]. Studies have shown that taking carbohydrate supplements before and between training sessions can significantly increase training volume and increase muscle glycogen synthesis [41]. Therefore, young adults who regularly participate in resistance exercise should still pay attention to carbohydrate intake to ensure better muscle strength performance.

Interestingly, in the present study, we observed that resistance exercise had the strongest association with B-vitamin intake. Participants’ weekly duration of resistance exercise was positively associated with intake of vitamins B2, B3, B5, B6, and B12. B vitamins are an important group of vitamins that perform an essential role in cell function [42]. There is growing evidence that there may be a relationship between vitamin B intake and muscle strength. Vitamin B6 deficiency is suggested to possibly affect distal motor neurons and may be associated with motor function loss [43]. Reduced vitamin B12 levels may also be a risk factor for muscle weakness and spasticity [44]. A study of diabetic patients over the age of 65 showed that levels of folate (vitamin B9) were positively associated with grip strength and leg strength [45]. Since several B vitamins act as cofactors in muscle synthesis and are important neurotrophic agents, B-vitamin deficiency may have an impact on muscle, nerve, and neuromuscular connections [46]. Dolopikou et al. [47] determined that elderly people taking vitamin B3 supplements can experience enhanced muscle strength and reduced fatigue. Therefore, individuals who are regularly involved in resistance exercise may need to consume more B vitamins to meet the needs of their body functions. The results of this study could also support this hypothesis, with the intake of most B vitamins increasing with the time spent with resistance exercise. However, it is worth noting that the intake of vitamin B1 and folate were not associated with resistance exercise. This may be strongly attributed to the daily dietary habits of the participants. We observed that those participants in this study who spent more time in resistance exercise tended to consume more protein in their daily diet. As foods rich in high-quality protein such as red meat, eggs, and milk are also dietary sources of vitamins B2, B3, B5, B6, and B12 [33], the intake of these B vitamins may increase with protein intake. However, the main food sources of vitamin B1 and folate were whole-grain products, rice bran, and green vegetables rather than meat [33]. Nevertheless, considering that resistance exercise was not associated with carbohydrate and fiber intake in this study, vitamin B1 and folate intake may have been relatively inadequate in participants who spent more time in resistance exercise. This also indicates that this group of participants should emphasize the intake of whole-grain products and green vegetables to ensure a more complete intake of B vitamins, which may be beneficial for their exercise performance and post-exercise recovery. 

In our analysis, of the association between time spent in exercise of all types and nutrient intake, time spent in aerobic exercise was positively associated with fiber and pectin intake. Pectin is a water-soluble fiber whose main food source is the tissue of plants [48]. Pectin, as well as other types of fiber, have positive effects on human health. However, consumption of beverages rich in fiber (including pectin) may reduce energy intake and appetite [49]. Dietary fiber intake may also have an effect on exercise capacity. Okamoto et al. [50] suggest that short-chain fatty acids produced by intestinal flora through dietary fiber may significantly improve the duration of endurance exercise. This may explain why aerobic exercise time was positively associated with total fiber and pectin intake in this study. Of course, there is another possible explanation, namely the fact that participants who spend more time in aerobic exercise prefer to consume fiber-rich foods. The motivation for this dietary behavior may stem from a variety of factors such as weight loss, keeping in shape, or practicing a healthier lifestyle. Several studies have shown that high fiber intake is inversely associated with weight gain [51] and that aerobic exercise combined with a high fiber diet has a beneficial effect on fat and weight loss, and can reduce muscle loss to some extent [52]. In this study, we also observed that time spent in aerobic exercise was negatively associated with the percentage of total calories from protein and that participants who performed aerobic exercise for more than 40 min per week were more likely to consume lesser amounts of vitamin B12 than suggested by the Dietary Reference Intake value. This may imply that the more frequently participants performed aerobic exercise, the less protein they tended to consume. In addition, since the main source of vitamin B12 is animal food [33], a relative decrease in protein intake is likely to lead to insufficient intake of vitamin B12. Therefore, this study recommends that participants who regularly perform aerobic exercise should pay attention to protein intake, while those who are strict vegetarians should pay attention to additional vitamin B12 supplementation besides the intake of plant-based protein. Additionally, this study notes that the time spent in aerobic exercise was positively associated with vitamin B6 intake, but participants who walked for more than 40 min per week were more likely to be deficient in vitamin B6 intake, which is not meeting the dietary reference intake. Interestingly, walking style tended to be negatively associated with almost all B vitamin intake, which could correlate with the metabolism of B vitamins in the body. Vitamin B2 is also known as riboflavin, which exists in the form of flavin mononucleotide (FMN) and flavin adenine dinucleotide (FAD), and is the cogroup of the oxidoreductase flavin protein. Flavin coenzymes play an important role in many REDOX cycles that depend on nicotinamide coenzymes (NADH and NADPH). However, vitamin B3 is the main component of NAD+ and NADP+. Vitamin B5 can crease the affinity of CoA which is the main active form of Vitamin B5. Vitamin B6 is a coenzyme of various enzymes involved in amino acid metabolism. Vitamin B12 converts methylmalonyl-CoA into succinyl-CoA in the body, which is involved in the tricarboxylic acid cycle. Succinyl-CoA is involved in hemoglobin synthesis. Metabolism is part of the aerobic process. Low-intensity walking was supplied to energy by aerobic metabolism. The results suggest that people who engage in less-intense walking have lower B vitamin intake and should pay more attention to additional supplements. The remaining types of exercise, such as sports exercise and other structured physical activities, have also been determined to be associated with fiber and pectin intake. The possible reasons and underlying mechanisms behind these associations may be related to the metabolism of sugar, fat and protein and further in-depth studies are needed in the future.

Our results also reveal the association between total exercise time and the intake of different nutrients. The longer the total exercise time, the higher the percentage of total calories from fat and protein, and the lower the percentage of total calories from carbohydrates. However, total exercise time did not show a significant negative association with the absolute amount of carbohydrate intake. Thus, this suggests that as total exercise time increases, carbohydrate intake does not increase proportionally, but rather decreases in relative amounts. Low-carbohydrate diets may have some benefits for weight loss, preventing diabetes and promoting healthy aging [53,54]. Brouns et al. [53] concluded that a non-ketogenic diet of 100–150 g of carbohydrate per day is more practical for weight loss and diabetes prevention. A prospective cohort study of 135,000 participants also showed that nutritive carbohydrate intake was associated with increased mortality rates [55]. It is important to note, of course, that many of the potential mechanisms of low-carbohydrate diets require further research and that strict low-carbohydrate diets (20–50 g per day) may also have some risks and adverse effects and should therefore be considered with caution [39]. A study on the association between carbohydrate intake and mortality detrmined that both high and low percentages of carbohydrate diets were associated with increased mortality, with minimal risk observed at 50–55% carbohydrate intake [56]. However, Feinman et al. [57] believed that Seidelmann et al.’s study [56] conclusions might have a risk of restricting patient choices, inhibiting future research, and impeding the advancement of public health if taken as a basis for recommendations. In this study, all participants did not severely restrict their carbohydrate intake, all met the dietary reference intake for carbohydrates and there was no association between total exercise time and the absolute amount of carbohydrate intake. Overall, therefore, this may support the idea that with increasing total exercise time, the ratio of carbohydrate, protein, and fat intake appears to be more beneficial for weight loss and health. Moreover, we observed a positive correlation between total exercise time and intake of vitamins B2, B5, and B12, and that participants with relatively sufficient total exercise time were more likely to consume fiber and vitamin B5 that met the dietary reference intake. Fiber is particularly noteworthy, and considering the benefits of fiber intake and the fact that only 9.1% of participants in this study consumed the recommended amount of fiber. It could also be possible that active people are more conscious of their diet and, therefore, choose healthier foods.

In this study, the association between different exercise types and nutrient intake was explored. The result could provide some new insights and references for future exercise and nutrition intervention strategies. However, this study also has some limitations that should be considered when interpreting the results. First, the results of the study cannot be generalized to the overall population as the majority of participants were young adults. Second, dietary intake as well as the types of exercise participants performed were self-reported. Even though best practice approaches have been taken to enhance the accuracy of the diet recalls (e.g., portion size training, visual aids during the interview, prompting), there remains a risk of misrepresentation of habitual dietary intake. Similarly, specific types of exercise that were not included in the questionnaire may have been missed. Third, the study was limited by the cross-sectional design, which did not allow to determine causal relationships. Future prospective studies with larger samples may be needed to deeply investigate the effects of different exercise types on nutrient intake.

## 5. Conclusions

The results showed an association between various exercise types and specific nutrients. It may be worthwhile to point out the negative association of exercise with CHO intake, which may need to be watched more closely in active young adults. In addition, the supplementation of B vitamins and pectin may be beneficial for their exercise performance and post-exercise recovery.

## Figures and Tables

**Table 1:** Baseline characteristics of participants.

	All Participants (*n =* 427)	Women (*n =* 217)	Men (*n =* 210)
Age (years)	27.65 ± 3.78	27.81 ± 3.70	27.47 ± 3.87
% European	284 (66.5%)	141 (65.0%)	143(68.1%)
% Employed for Wages	224 (52.5%)	121 (55.8%)	103 (49.0%)
% Married	139 (32.6%)	64 (29.5%)	75 (49.0%)
Height (cm)	171.10 ± 9.45	164.57 ± 6.49	177.84 ± 6.98
Weight (kg)	75.30 ± 14.01	69.35 ± 12.71	81.44 ± 12.58
BMI (kg/m^2^)	25.65 ± 3.87	25.58 ± 4.28	25.71 ± 3.40
Fat Mass (kg)	21.50 ± 8.62	24.09 ± 8.33	18.80 ± 8.09
Lean Mass (kg)	51.36 ± 10.77	43.26 ± 5.99	59.76 ± 7.78
Body Fat (%)	28.40 ± 8.98	33.97 ± 6.66	22.61 ± 7.26
Light PA (min/day)	216.18 ± 58.44	235.88 ± 58.56	195.83 ± 50.98
MVPA (min/day)	135.58 ± 77.15	112.42 ± 65.21	159.51 ±81.26

Abbreviations: BMI: body mass index; PA: physical activity; MVPA: moderate to vigorous physical activity.

**Table 2:** Descriptive characteristics of exercise time for different exercise types.

	Exercise Time	Exercise Time < 40 min	Exercise Time > 40 min
	Median (IQR)	*n* (%)	*n* (%)
Resist EX (min/week)	40.00 (120.00)	219 (51.3%)	208 (48.7%)
Aerobic EX (min/week)	60.00 (135.00)	190 (44.5%)	237 (55.5%)
Sports EX (min/week)	00.00 (60.00)	301 (70.5%)	126 (29.5%)
Walking (min/week)	40.00 (90.00)	219 (51.3%)	208 (48.7%)
Other EX (min/week)	00.00 (120.00)	233 (54.6%)	194 (45.4%)

Abbreviations: EX: exercise.

**Table 3:** Intake of different nutrients for different exercise times.

	Median (IQR)	Exercise Time > 240 min	Exercise Time < 240 min
Total EX (Min/Week)	240.00 (350.00)	216 (50.6%)	211 (49.4%)
	Nutrient Intake	Achieve DRI	DRI Not Achieved
	Median (IQR)	*n* (%)	*n* (%)
Fat (g/day) ^a^	71.47 (43.41)	—	—
CHO (g/day)	231.85 (103.23)	427 (100.0%)	0 (0.0%)
PRO (g/day)	78.86 (38.83)	396 (92.7%)	31 (7.3%)
Sugar (g/day) ^a^	88.65 (52.94)	—	—
Fiber (g/day)	17.18 (10.51)	39 (9.1%)	388 (90.9%)
Pectin (g/day) ^a^	2.05 (1.55)	—	—
Vitamin B1 (mg/day)	1.64 (0.85)	365 (85.5%)	62 (14.5%)
Vitamin B2 (mg/day)	1.97 (1.07)	395 (92.5%)	32 (7.5%)
Vitamin B3 (mg/day)	24.42 (12.97)	399 (93.4%)	28 (6.6%)
Vitamin B5 (mg/day)	5.06 (3.27)	221 (51.8%)	206 (48.2%)
Vitamin B6 (mg/day)	1.88 (1.10)	364 (85.2%)	63 (14.8%)
Vitamin B9 (*μ*g/day)	422.11 (230.79)	233 (54.6%)	194 (45.4%)
Vitamin B12 (*μ*g/day)	4.15 (3.39)	348 (81.5%)	79 (18.5%)

Note: ^a^ Lack or absence of uniform dietary reference intake standards. Abbreviations: EX: exercise; CHO: carbohydrate; PRO: protein; DRI: dietary reference intakes.

**Table 4:** The associations between the time of participation in different types of exercise and nutrient intake.

	Exercise (Min/Week)											
Dependent	Resist EX ^1^		Aerobic EX ^1^		Sports ^1^		Walking ^1^		Other Structured PA ^1^		Total EX ^2^	
Variable	*β*	*p*	*β*	*p*	*β*	*p*	*β*	*p*	*β*	*p*	*β*	*p*
Fat	0.087	0.109	0.053	0.265	−0.055	0.263	−0.049	0.300	0.057	0.269	0.091	0.053
CHO	−0.078	0.160	0.086	0.075	0.008	0.869	0.005	0.923	−0.101	0.056	−0.061	0.209
PRO	0.157	**0.003**	−0.037	0.409	−0.043	0.359	−0.045	0.323	0.012	0.810	0.064	0.155
PCT-Fat	0.093	0.099	0.013	0.786	−0.017	0.737	−0.018	0.722	0.100	0.064	0.116	**0.018**
PCT-CHO	−0.166	**0.002**	0.013	0.789	0.024	0.630	0.043	0.371	−0.093	0.077	−0.142	**0.003**
PCT-PRO	0.191	**0.001**	−0.130	**0.008**	0.015	0.770	−0.017	0.724	0.096	0.072	0.126	**0.012**
Sugar	−0.043	0.457	0.073	0.148	0.040	0.449	0.039	0.442	−0.064	0.246	0.006	0.911
Fiber	0.020	0.727	0.129	**0.009**	−0.140	**0.007**	−0.061	0.218	0.084	0.120	0.076	0.127
Pectin	0.032	0.567	0.115	**0.020**	−0.194	**0.000**	−0.047	0.349	0.131	**0.016**	0.078	0.120
Vitamin B1	0.018	0.744	0.062	0.192	−0.007	0.899	−0.038	0.426	−0.067	0.200	−0.006	0.903
Vitamin B2	0.138	**0.009**	0.081	0.080	0.029	0.553	−0.037	0.421	−0.037	0.470	0.138	**0.003**
Vitamin B3	0.141	**0.008**	0.015	0.738	−0.037	0.435	−0.009	0.848	−0.062	0.218	0.048	0.296
Vitamin B5	0.161	**0.003**	0.076	0.107	0.005	0.916	−0.069	0.145	−0.010	0.851	0.155	**0.001**
Vitamin B6	0.143	**0.010**	0.101	**0.037**	−0.087	0.082	−0.041	0.403	−0.027	0.609	0.088	0.070
Vitamin B9	0.035	0.529	0.068	0.164	−0.043	0.397	0.001	0.989	−0.031	0.570	0.026	0.596
Vitamin B12	0.149	**0.006**	−0.024	0.609	0.094	0.057	−0.016	0.741	−0.021	0.690	0.130	**0.006**

Note: All models were controlled for sex, age, ethnicity, employment status, and marital status. All regression coefficients were standardized. ^1,2^ indicate separate linear regression models. Bold font indicates statistical significance (*p* < 0.05). Abbreviations: EX: exercise; CHO: carbohydrate; PRO: protein; PCT-fat: percentage of total calories from fat; PCT-CHO: percentage of total calories from carbohydrate; PCT-PRO: percentage of total calories from protein; DRI: dietary reference intakes.

**Table 5:** Odds ratios of achieving DRI for nutrient intake in the study participants.

			Nutrients			
Independent	Fiber	Vitamin B1	Vitamin B5	Vitamin B6	Vitamin B9	Vitamin B12
Variable	OR (95%CI)	OR (95%CI)	OR (95%CI)	OR (95%CI)	OR (95%CI)	OR (95%CI)
Resist EX ^1^						
<40 min/week	—	—	— Ref. —		—	—
>40 min/week	1.842 (0.827, 4.102)	1.251 (0.668, 2.343)	1.464 (0.925, 2.317)	1.707 (0.893, 3.262)	1.095 (0.699, 1.715)	1.128 (0.633, 2.011)
Aerobic EX ^1^						
<40 min/week	—	—	— Ref. —		—	—
>40 min/week	1.458 (0.676, 3.145)	0.734 (0.399, 1.350)	1.023 (0.660, 1.585)	1.068 (0.585, 1.949)	1.032 (0.672, 1.583)	0.506 * **(0.285, 0.899)**
Sports EX ^1^						
<40 min/week	—	—	— Ref. —		—	—
>40 min/week	0.545 (0.229, 1.297)	0.735 (0.380, 1.421)	1.031 (0.643, 1.655)	1.080 (0.527, 2.217)	0.760 (0.478, 1.208)	1.643 (0.840, 3.212)
Walking ^1^						
<40 min/week	—	—	— Ref. —		—	—
>40 min/week	1.900 (0.901, 4.009)	0.940 (0.521, 1.693)	0.976 (0.638, 1.495)	0.461 * **(0.248, 0.856)**	0.739 (0.488, 1.118)	1.137 (0.658, 1.964)
Other EX ^1^						
<40 min/week	—	—	— Ref. —		—	—
>40 min/week	1.754 (0.798, 3.854)	0.995 (0.534, 1.853)	1.285 (0.812, 2.034)	1.326 (0.700, 2.513)	1.249 (0.799, 1.951)	1.057 (0.594,1.881)
Total EX ^2^						
<240 min/week	—	—	— Ref. —		—	—
>240 min/week	3.023 * **(1.403, 6.514)**	0.878 (0.489, 1.577)	1.616 * **(1.061, 2.462)**	1.441 (0.795, 2.613)	1.108 (0.734, 1.673)	1.182 (0.691, 2.023)

Note: All models controlled for sex, age, and ethnicity. ^1,2^ indicate separate logistic regression models; * and bold font indicates statistical significance (*p* < 0.05). Abbreviations: EX: exercise; OR: odds ratio; CI: confidence interval; Ref: reference; DRI: dietary reference intakes.

## Data Availability

The data presented in this study are available on request from the corresponding author. The data are not publicly available due to confidentiality.
